# Molecular epidemiology of coagulase-negative *Staphylococcus* carriage in neonates admitted to an intensive care unit in Brazil

**DOI:** 10.1186/1471-2334-13-572

**Published:** 2013-12-05

**Authors:** Yves Mauro Ternes, Juliana Lamaro-Cardoso, Maria Cláudia Porfirio André, Vicente Porfírio Pessoa, Maria Aparecida da Silva Vieira, Ruth Minamisava, Ana Lúcia Andrade, André Kipnis

**Affiliations:** 1 Institute of Tropical Pathology and Public Health, Federal University of Goiás, Goiânia, GO, Brazil; 2 Children’s Hospital, Goiânia, Brazil; 3 Pontifical Catholic University of Goiás, Goiânia, GO, Brazil; 4 School of Nursing, Federal University of Goiás, Goiânia, GO, Brazil

**Keywords:** Coagulase-negative *Staphylococcus*, *mec*A, SCC*mec*, Neonatal intensive care units, Neonates

## Abstract

**Background:**

Nasal colonization with coagulase-negative *Staphylococcus* (CoNS) has been described as a risk factor for subsequent systemic infection. In this study, we evaluated the genetic profile of CoNS isolates colonizing the nares of children admitted to a neonatal intensive care unit (NICU).

**Methods:**

We assessed CoNS carriage at admittance and discharge among newborns admitted to a NICU from July 2007 through May 2008 in one of the major municipalities of Brazil. Isolates were screened on mannitol salt agar and tryptic soy broth and tested for susceptibility to antimicrobials using the disc diffusion method. Polymerase chain reaction (PCR) was used to determine the species, the presence of the *mec*A gene, and to perform SCC*mec* typing. *S. epidermidis* and *S. haemolyticus* isolated from the same child at both admission and discharge were characterized by PFGE.

**Results:**

Among 429 neonates admitted to the NICU, 392 (91.4%) had nasal swabs collected at both admission and discharge. The incidence of CoNS during the hospitalization period was 55.9% (95% confidence interval [CI]: 50.9-60.7). The most frequently isolated species were *S. haemolyticus* (38.3%) and *S.epidermidis* (38.0%). Multidrug resistance (MDR) was detected in 2.2% and 29.9% of the CoNS isolates, respectively at admittance and discharge (p = 0.053). The *mec*A gene was more prevalent among strains isolated at discharge (83.6%) than those isolated at admission (60%); overall, SCC*mec* type I was isolated most frequently. The length of hospitalization was associated with colonization by MDR isolates (p < 0.005). Great genetic diversity was observed among *S. epidermidis* and *S. haemolyticus*.

**Conclusions:**

NICU represents an environment of risk for colonization by MDR CoNS. Neonates admitted to the NICU can become a reservoir of CoNS strains with the potential to spread MDR strains into the community.

## Background

Coagulase-negative *Staphylococcus* (CoNS) are the most common bacteria associated with neonatal healthcare-associated infections [1]. Colonization with CoNS is a risk factor associated with infection among neonates [2]. The neonatal hospital population has several characteristics that favor the development of infections; primarily, these include the immature immune system of the neonates, the use of invasive procedures, and aggressive antibiotic therapy protocols [3,4]. Consequently, CoNS strains play an important role in the environment of neonatal intensive care units (NICUs) [5].

Rates of methicillin resistance up to 80% have been observed among CoNS isolated from bloodstream infections in NICU patients [1]. Resistance to other classes of antibiotics has also emerged in the last few decades [6,7]. *S.epidermidis,* considered as the main reservoirs for multidrug resistant (MDR), have been isolated from the skin of patients and health care staff, medical equipment, healthcare professionals’ clothing, and hospital surfaces [8]. *S. epidermidis* is the predominant CoNS species in infections associated with immune deficiency, catheters, and other invasive procedures [9,10]; in most cases, infection occurs due to migration of the microorganism from the skin to the vascular catheter insertion point, which favors hematogenic dissemination and infection [4].

Infections caused by CoNS are related to a significant increase in acute neonatal mortality; in some cases, they may be associated with the development of respiratory complications [11,12]. The identification of nasal colonization by CoNS could be useful for the prevention of future infections. [13].

Most of the studies on CoNS nasal colonization in neonates were conducted in the1990s [2] and showed a colonization prevalence ranging from 13% to 56% [14,15]. A recent study investigated early colonization by CoNS in 46 preterm neonates at the NICU and found that 55% of the neonates were colonized within the first 3 days of hospitalization [12].

In Latin America, information regarding the characteristics of CoNS carriage in neonates as well as the relationship between colonizing and invasive CoNS isolates is scarce [16]. In this study, we assessed different species of CoNS and antibiotic susceptibility of isolates colonizing the nares of children admitted to a NICU of a major city located in Central Brazil. Potential risk factors for colonization and the genetic relatedness among the CoNS strains isolated at admission and discharge were also evaluated.

## Methods

### Patient enrollment and sample collection

This prospective study was carried out from July 2007 through May 2008 in the NICU of Hospital da Criança (26 neonatal beds), located in Goiania (**~**1,300,000 inhabitants). All children admitted to the NICU during the study period were eligible for nasal swab collection. The investigation was approved by the Regional Ethical Committee of Hospital Materno Infantil (CEP-HMI #006/07), and written consent was obtained from the neonates’ parents. A nasal swab (Copan®, CA, USA) was collected from the neonates at admittance and discharge, and transported in Stuart’s medium to the Microbiology Laboratory of the Federal University of Goiás.

### Risk factors

Potential risk factors for CoNS colonization, which were obtained from medical records by physicians and nurses, included type of birth, sepsis occurrence, prematurity, age (days) at hospitalization, antimicrobial use during hospitalization, low birthweight, length of hospitalization (days), use of continuous positive airway pressure (CPAP), gender, and comorbidities (chronic, genetic, infectious diseases, and fetal malformation). The criteria for sepsis definition followed the Centers for Disease Control and Prevention (CDC) guidelines [17] with local adaptations.

### Microbiological procedures

The nasal swabs were inoculated onto mannitol salt agar and one suggestive colony from each patient was submitted to screening tests. Each colony was identified by standard methods [18].

### Susceptibility tests

CoNS isolates were submitted to a disk diffusion susceptibility test with the following antibiotics: oxacillin (1 μg), cefoxitin (30 μg), erythromycin (15 μg), clindamycin (2 μg), quinupristin-dalfopristin (15 μg), rifampicin (15 μg), ciprofloxacin (5 μg), tetracycline (30 μg), sulfamethoxazole-trimethoprim (23.75/1.25 μg), linezolid (30 μg), and penicillin (10 μg) (Oxoid®, Basingstoke, England). Inhibition halos were interpreted according to the Clinical Laboratory and Standards Institute (CLSI) guidelines [19]. The D test for macrolide-lincosamide-streptogramin B (MLSb) resistance was performed according to Fiebelkorn et al. [20]. Resistance to at least four classes of antibiotics was defined as MDR [21]. Vancomycin and teicoplanin susceptibilities were not tested.

### Identification of CoNS species

DNA was extracted from the isolates identified as CoNS according to the method of van Embden et al. [22]. Internal transcribed spacer (ITS) PCR was performed according to Couto et al. [23] to identify the staphylococcal species. Reference strains for *S. epidermidis* (DEN125)*, S. haemolyticus* (ICE145)*, S. warneri*(DEN157)*, S. lugdunensis* (ICE187)*, S. saprophyticus* (DEN177), *S. caprae* (COB-25), *S. cohnii* (ITL152), *S. simulans (*AGT121), and *S. carnosus* (TAW88) were kindly provided by Dr. Hermínia de Lencastre (Laboratory of Molecular Genetics, Institute for Chemical and Biological Technology, Nova Lisboa University, Oeiras, Portugal) and used as controls. The PCR product of each strain was compared to those of the reference strains. A species was assigned when a perfect match of the PCR products of a particular sample with that of the reference strain was obtained [23].

### SCC*mec* typing

PCR was performed as previously described by Geha et al. [24] to ascertain the presence of the *mec*A gene for all CoNS strains. Staphylococcal cassette chromosome *mec* (SCC*mec*) typing was performed by multiplex PCR [25]. The regions identified were as follows: type I (J1 region); type II (J1 and J3 regions, *ccr* complex, and *mec* complex); type III (J1 and J3 regions and *mec* complex); type IV (J3 region and *ccr* complex); type V (J1 region and *ccr* complex); and type VI (J3 region). PCR was performed in a thermocycler (Biocycler®, Zhejiang, China), and the PCR products were resolved by electrophoresis on a 1.5% agarose gel stained with ethidium bromide. A Gel Doc XR System (Bio-Rad® Laboratories, Hercules, CA, USA) was used for visualization and analysis. The following *Staphylococcus aureus* strains (kindly provided by Dr. Herminia de Lencastre, ICBT, UNL, Oeiras, Portugal) were used as controls for SCC*mec* typing: COL (type I); N316 (type II); ANS46 (type III); MW2 (type IV); and WIS (type V). Strains harboring the *mec*A gene that did not produce amplification of any SCC*mec* gene were assigned as nontypeable (NT).

### Multilocus sequence typing (MLST) of *Staphylococcus epidermidis*

Determination of MLST was performed by sequencing the PCR amplicons of the following housekeeping genes: shikimate dehydrogenase (*aroE*), carbamate kinase (*arc*C), ABC transporter (*gtr*), DNA mismatch repair protein (*mutS*), pyrimidine operon regulatory protein (*pyr*R), triphosphate isomerase (*tpiA*), and acetyl coenzyme A acetyltransferase (*yqiL*). Primers used for amplification of the seven housekeeping genes were those recommended by the unified MLST scheme (http://sepidermidis.mlst.net/misc/info.asp) that employs the internal fragments of *arcC* and *aroE* from the Wisplinghoffet al. MLST scheme [26], *gtr*, *mutS* and *pyrR* loci from the Thomas et al. MLST scheme [27] and sequences from the *tpiA* and *yqiL* genes used by the Wang et al. MLST scheme [28]. Sequencing of purified amplicons was performed on an ABI 3130 XL genetic analyzer (Applied Biosystems, Foster City, CA, USA). Sequences obtained were assigned allele numbers that were used to generate sequence types (STs) for each isolate.

### Pulsed field gel electrophoresis (PFGE) analysis

The genetic relatedness of the most prevalent species (*S. epidermidis* and *S. haemolyticus*) was determined by PFGE according to Chung et al. [29]. Band patterns were interpreted based on the criteria described by Tenover et al. [30], and analyses were performed using BioNumerics® software version 5.0 (Applied Maths, Kortrijk, Belgium). The resulting dendrogram was constructed using the unweighted pair group method for arithmetic averages and the Dice band-based similarity coefficient. Isolates were defined as epidemiologically related (same cluster) if they shared ≥80% similarity on the dendrogram [31].

### Data analysis

All data were analyzed with SPSS® software version 18.0 (Chicago, USA). Neonates carrying CoNS at admission were removed from the analysis for incidence rate calculations. The susceptibility profiles of the isolates at admission and discharge were analyzed to evaluate the progression of drug resistance among CoNS during the period of hospitalization. Univariate analyses were performed with the Chi-square and Mann–Whitney tests, and relative risk for MDR CoNS carriage, with the respective 95% confidence intervals (95% CI), was calculated. Odds ratio and 95% CI was used in multiple logistic regression model to measure the effect of the exposure variables on MDR CoNS carriage using backward stepwise elimination. The Hosmer–Lemeshow statistical test was used to determine the goodness-of-fit of the logistic regression model. P-values less than 0.05 were considered significant for all analyses.

## Results

A total of 429 neonates were admitted to the NICU during the study period. Nasal swabs were collected from 392 (91.4%) neonates at both admission and discharge. The frequency of nasal carriage by CoNS was 69.9% (95% confidence interval [CI]: 65.2-74.3). The incidence of colonization by CoNS in the neonatal cohort during the hospitalization period was 55.9% (95% CI: 50.9-60.7).

**Figure 1:**
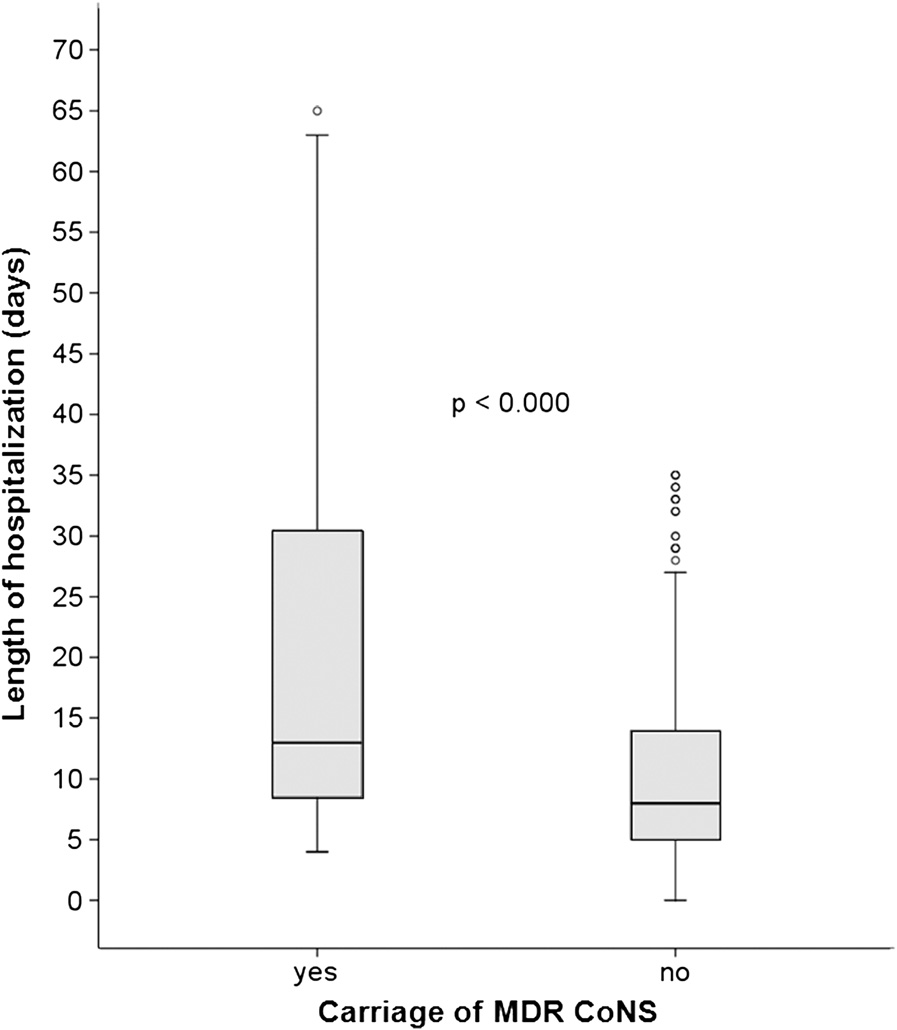
**Length of hospitalization among neonates 422 admitted to Neonatal intensive care units according to CoNS multidrug resistance.** The line across the box indicates the median values. The circles represent the outliers for length of stay.

The antibiotics associated with MDR were oxacillin/cefoxitin, erythromycin, clindamycin, ciprofloxacin, tetracycline, and sulfamethoxazole-trimethoprim. There was a significant increase in the frequency of MDR CoNS isolates at discharge compared with the frequency at admission (29.9% and 2.2%, respectively, p = 0.053). The frequencies of resistance to all antimicrobials tested, except for tetracycline, were higher among the CoNS isolated at discharge compared with the CoNS isolated at admission. Tetracycline resistance decreased among the CoNS from admission to discharge. Nasal carriage of MDR CoNS was significantly associated with length of hospitalization (Figure 1).

The prevalence of β-lactam antibiotic (cefoxitin and oxacillin) resistance among 45 CoNS isolates at admission was 57.8% and increased to 89.3% among 244 isolates at discharge (p = 0.165); two neonates were colonized by CoNS with a MLSb-resistant phenotype (one at admission and one at discharge). The *mec*A gene was detected at frequencies of 60.0% and 83.6% at admission and at discharge, respectively. Antimicrobial susceptibility for isolates from twenty-four children was not performed due to failure to recover viable culture. The frequencies of the *mec*A gene and bacterial resistance among CoNS isolates were higher (p = 0.296) at discharge compared with admission (Table 1).

**Table 1:** **Cefoxitin resistance and presence of the *mec*** **A gene among CoNS nasal isolates obtained from neonates in the NICU at admission and discharge from July 2007 through May 2008**

**Variables**	**Coagulase-negative *Staphylococcus *** **isolates**			
	**Admission (N = 45)**		**Discharge (N = 244)**	
	**n**	**%**	**N**	**%**
Cefoxitin				
Resistant	26	57.8	218	89.3
Susceptible	19	42.2	26	10.7
*mec*A genotype				
Positive	27	60.0	204	83.6
Negative	18	40.0	40	16.4
Multidrug resistance^a^				
Yes	1	2.2	73	29.9
No	44	97.8	171	70.1

^a^Multidrug resistance was considered as resistance to at least four classes of antibiotics.

SCC*mec* typing revealed similar frequencies of types I, and IV at both admission and discharge. Types I, II, and III accounted for the majority of SCC*mec* identified in this study. Several CoNS isolates presented more than one SCC*mec* type (45.8% of the isolates from admission and 46.5% from discharge) (Table 2 and Additional file 1: Table S1).

**Table 2:** **SCC** ***mec *** **frequencies at admission and discharge, among the most prevalent *mec*** **A containing strains isolated from neonates from July 2007 through May 2008**

**SCC** ***mec *** **type**	***S. epidermidis *** **(%)**		***S. haemolyticus *** **(%)**		***S. capitis *** **(%)**		***S. warneri *** **(%)**	
	Adm*	Disch**	**Adm**	**Disch**	**Adm**	**Disch**	**Adm**	**Disch**
**I**	1 (8.3)	7 (9.6)	3 (25.0)	56 (68.3)	0	0	0	1 (50)
**II**	0	1 (1.4)	0	0	0	0	0	0
**III**	0	4 (5.5)	1 (8.3)	1 (1.2)	0	2 (11.8)	0	0
**IV**	3 (25.0)	9 (12.3)	0	1 (1.2)	0	0	0	0
**V**	1 (8.3)	1 (1.4)	0	0	0	0	0	0
**2 types**	4 (33.3)	35 (47.9)	2 (16.7)	7 (8.6)	0	15 (88.2)	0	0
**3 or more**	1 (8.3)	12 (16.4)	4 (33.3)	11 (13.4)	0	0	0	0
Non typeable***	2 (16.8)	4 (5.1)	2 (16,7)	6 (7.3)	0	0	0	1 (50)
**Total**	12	73	12	82	0	17	0	2

*Adm: Admission.

**Disch: Discharge.

***Nontypeable: Defined as strains harboring the *mec*A gene, that did not produce amplification of any SCC*mec* gene.

*S. epidermidis* (38.3%) and *S. haemolyticus* (38.0%) were the most frequent species colonizing the neonates followed by *S. capitis* (4.7%), *S. warneri* (4.4%), *S. kloosii* (1.4%), *S. caprae* (1.4%), *S. schleiferi* (1.1%), *S. hominis* (0.7%), *S. lugdunensis* (0.7%), and *S. saprophyticus* (0.4%). *S. epidermidis* was primarily observed at admission, whereas *S. haemolyticus* predominated at discharge. Twenty-four isolates (8.2%) showed ITS-PCR patterns that differed from those of the reference strains and could therefore not be assigned to a species.

**Figure 2:**
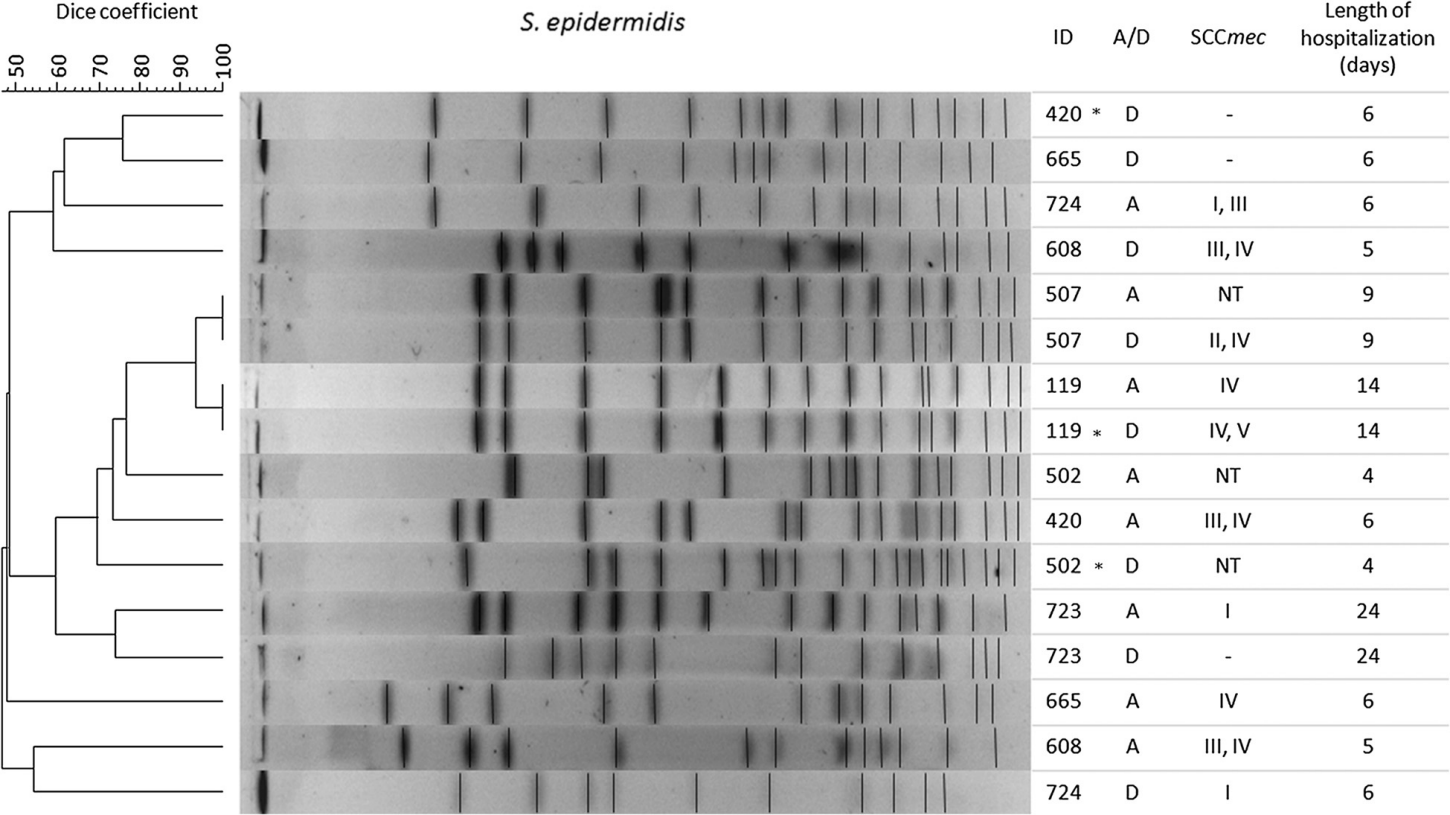
**PFGE dendrogram of *S. epidermidis.*** Genetic similarity of eight isolates from eight patients at hospital admission (A) and discharge (D). NT: Nontypeable; -: not applicable, *mec*A negative; *: strain with MDR phenotype.

**Figure 3:**
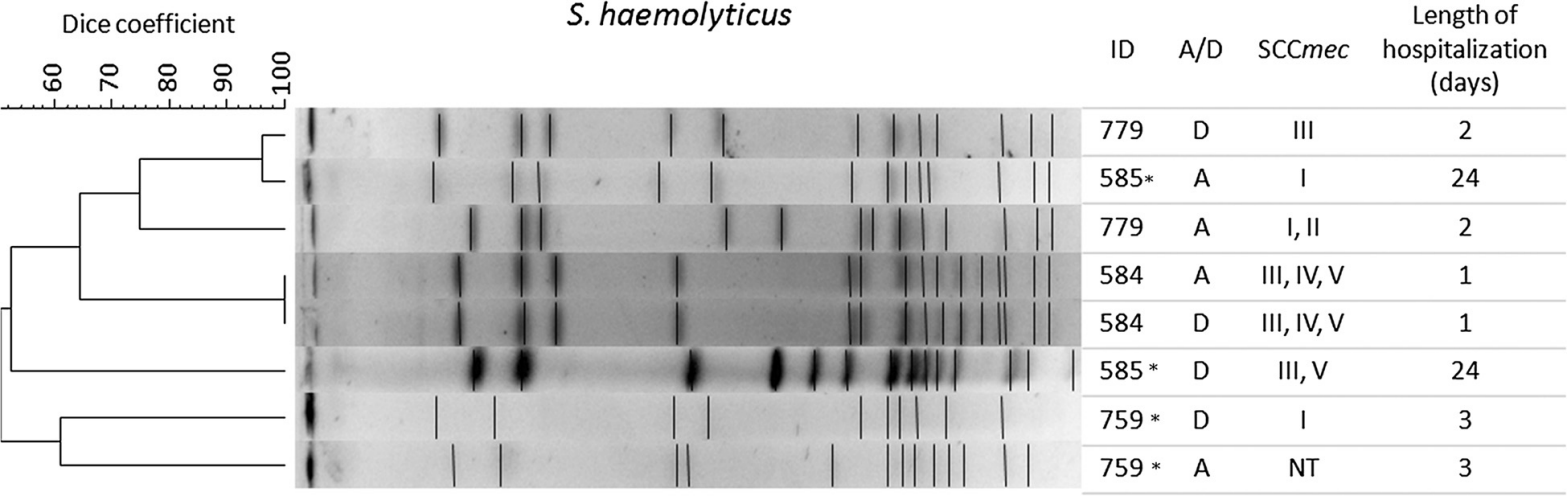
**PFGE dendrogram of *S. haemolyticus.*** Genetic similarity isolates from four patients at hospital admission (A) and discharge (D). NT: Nontypeable; *: strain with MDR phenotype.

The persistence of the same strain within a particular individual during the hospitalization period was evaluated by PFGE for *S. epidermidis* and *S. haemolyticus,* which were the most prevalent species isolated in this study. *S. epidermidis* was isolated from eight patients and *S. haemolyticus* was isolated from four patients at both admission and discharge, and the PFGE dendrogram revealed significant genetic diversity between the strains (Figures 2 and 3). Two patients (#507 and #119) were colonized by the same *S. epidermidis* clone during their entire hospitalization period. One patient (#608) presented strains with a mixed SCC*mec* type (III/IV) at both admission and discharge despite the low similarity (<50%) between them. Only one patient (#584) was persistently colonized by the same *S. haemolyticus* clone, which can be explained by the short duration of hospitalization (one day). Two patients (#779-discharge, #585-admission) presented strains with different SCC*mec* types (III and I, respectively) despite the high similarity (>90%) between them. Among the studied risk factors, the length of hospitalization was the only significant variable (*p* < 0.001) associated with MDR CoNS colonization (Table 3).

**Table 3:** Risk factors for multidrug resistance in CoNS isolates among children admitted to the NICU in Brazil from July 2007 through May 2008

**Variables**	**Incident cases of MDR CoNS**^**a**^		**RR**	**95% CI**	***P *** **value**	**OR**^**b**^	**95% CI**	***P *** **value**
	**Yes**	**No**						
Sex								
Male	46	184	1.29	0.80-2.34	0.256			
Female	25	136						
Antimicrobial use								
Yes	62	241	2.42	1.10-5.37	0.011	0.51	0.21-1.26	0.144
No	6	65						
Low birthweight								
Yes	43	162	1.26	0.88-2.09	0.162			
No	28	153						
Age at hospitalization								
<24 hours	55	241	1.12	0.67-1.85	0.670			
≥24 hours	16	80						
Type of birth								
Caesarean	39	219	0.61	0.40-0.93	0.023	1.70	0.97-2.99	0.064
Vaginal	32	97						
CPAP^c^								
Yes	30	97	1.53	1.00-2.35	0.056	1.08	0.58-2.03	0.805
No	38	208						
Hospitalization^d^								
Median	9	2			< 0.05	0.97	0.96-0.98	0.000
Interquartile range	5-15	2-2						

^a^Multidrug-resistant (resistance to four or more classes of antimicrobials), coagulase-negative *Staphylococcus.*

^b^Odds Ratio adjusted for age at hospitalization and sex.

^c^Continuous positive airway pressure.

^d^Length of hospitalization (days).

The molecular characterization of eight *S. epidermidis* strains representatives of different PFGE patterns by MLST identified 7 STs. Two strains were assigned as ST 59, one as ST 81 and one as ST 86, while the other four strains were assigned new STs based on their allelic profiles and were single-locus variants (SLV) of STs 21, 130, 158,291, 351, 450, or 466 (Additional file 2: Table S2 and Additional file 3: Figure S1). The observed STs are included in the major clonal complex (CC2) of the *S. epidermidis* lineages.

## Discussion

Few investigations have been performed to examine CoNS neonatal colonization in NICU settings. We observed a lower incidence of CoNS colonization in neonates during hospitalization (55.9%) compared with Campeotto et al. [32], who observed a 66.1% incidence of colonization in nasal specimens from children admitted to the NICU. However, we collected samples only at admittance and discharge, whereas the cited study sampled the children weekly.

We found that the prevalence of MDR was increased among CoNS strains isolated at discharge, which could most likely be explained by the intensive selective pressure caused by antibiotic therapy administered to patients admitted to the NICU, as reported by others [33]. The increase of MDR prevalence could not be explained by an increase in frequency of colonization by *S. haemolyticus* alone, as susceptibility profile of *S. haemolyticus* isolated at admission showed that they were as susceptible as other species (data not shown). We also detected a high rate of antimicrobial resistance to β-lactam antibiotics (oxacillin and cefoxitin), which is conferred by the *mec*A gene. Similar to our results, studies of colonization and sepsis among hospitalized neonates have reported that most CoNS species carry the *mec*A gene and that microorganisms isolated from systemic diseases have even higher resistance rates [1,34]. Taken together, these observations support the hypothesis that CoNS may be a genetic repository of antibiotic resistance genes, particularly *mec*A, and may serve as a carrier and/or reservoir of antibiotic resistance, which increases the risk of staphylococcal infection in the hospital environment [35]. In Brazil, the use of tetracycline in pediatrics is very limited, which may explain the lower prevalence of tetracycline resistance among CoNS isolated at discharge in our findings.

Studies have shown that the frequency of SCC*mec* among CoNS is increasing due to the wide dissemination of the microorganism in hospital-acquired infections, which is associated with the potential genetic transfer of resistance elements among *Staphylococcus* spp. [36]. We observed a high frequency (86.7%) of SCC*mec* types I, II, and III among CoNS isolates at discharge (considering isolates with only one SCC*mec* type). Similar SCC*mec* type frequencies were observed among CoNS isolated from blood culture of patients attending a tertiary hospital in southern Brazil [37]. Data regarding SCC*mec* typing of CoNS carriage among neonates are scarce in the literature, and caution must be taken when making comparisons of our findings. These types are commonly related with healthcare-associated methicillin-resistant *Staphylococcus aureus* (MRSA) [38], although it is not clear what SCC*mec* types are associated with particular CoNS species. Although CoNS isolates often present SCC*mec* types similar to those of *S.aureus*, they may also present unique, complex groups of SCC*mecs*[13], thereby acting as reservoirs for particular SCC*mec* genes and elements. The diverse SCC*mec* type profile among CoNS strains, as described previously [39], is due to the higher capacity for genetic transfer between the species. In addition, CoNS strains present a combination of SCC*mec* types, with SCC*mec* type IV being most frequently associated with other types of chromosomal cassettes. Due to the various combinations of different SCC*mec* types in the CoNS observed here and reported by others [40,41], there is aclear need to develop a unique typing system for this group of bacteria.

Although *S. epidermidis* has been reported as the most frequent CoNS species colonizing neonates, the most prevalent CoNS species colonizing the neonates at discharge in our study was *S. haemolyticus*. This observation could be related to the ability of *S. haemolyticus* to adapt to selective pressures, such as antimicrobials and biocides present in the hospital environment [8,42].

The high degree of genetic diversity among CoNS, as revealed by PFGE, and the presence of different SCC*mec* types at both admittance and discharge suggest that nasal colonization by these species is a very dynamic process in the NICU environment. Although a neonate is typically admitted to the hospital with a particular strain of *S. epidermidis*, there is a high probability that the nasal colonization will be replaced by a *Staphylococcus* species that is highly drug resistant and likely acquired from the hospital environment or professional staff [13]. The length of hospitalization could be associated with MDR CoNS, as a longer stay would result in greater exposure to these factors.

Although there are scarce data regarding CoNS colonization, blood culture-positive newborns have been associated with CoNS colonization [12]. Further studies are needed to address the effect of colonization with MDR CoNS on infection among neonates that are admitted to intensive care and undergo invasive procedures.

The identification of a single genetic lineage (CC2) comprising the majority of STs was showed worldwide [10]. The CC2 is considered the most prevalent complex in the nosocomial population of *S. epidermidis* and has been characterized by a high level of genetic diversity, an increased recombination/mutation rate, and a high number of SCC*mec* elements [10,43] as demonstrated in this study.

The present study has some limitations. Ideally, several nasal swabs should be collected during the neonatal hospitalization period to assess the dynamic characteristics of CoNS carriage status. Additionally, the currently available techniques for SCC*mec* characterization of CoNS are based on the *S. aureus* prototype. The SCC*mec* typing method used in this study is limited to the identification of certain region combinations, mainly J regions. It would be desirable to use entire nucleotide sequence to ascertain SCC*mec* elements and avoid possible misclassification [44].

## Conclusion

We have shown that children admitted to the NICU have a high rate of nasal colonization by CoNS with an increased frequency of MDR strains during the hospitalization period, primarily at discharge. In addition, we observed a high rate of SCC*mec* dissemination in the healthcare environment. An extended stay in the NICU could result in a greater risk for neonate colonization by MDR CoNS strains and consequently an increased risk of systemic infection by these microorganisms.

## Competing interests

The authors declare that they have no competing interests.

## Authors’ contributions

ALA and AK participated on the conception and design of the study, analysis and interpretation of data and drafting the manuscript. YMT and JLC participated in acquisition of data, molecular tests, interpreting results and revised the manuscript. MCPA participated in acquisition of data, molecular tests, and critically contributed to the draft of the manuscript. VPPJ and MASV participated in patients’ recruitment, supervision of all epidemiological data collection, interpretation of data and revising the manuscript. RM contributed on the study design, analysis and interpretation of epidemiological data, managing the database, and critically revised the manuscript. All authors read and approved the final version of the manuscript.

## Pre-publication history

The pre-publication history for this paper can be accessed here:

http://www.biomedcentral.com/1471-2334/13/572/prepub

## Supplementary Material

Additional file 1: Table S1 SCC*mec* types and frequencies at admission and discharges among strains with more than one type isolated from neonates from July 2007 through May 2008. Click here for file

Additional file 2: Table S2 MLST analysis from eight *S. epidermidis* strains isolated from neonates in this study. Sequencing analysis of each locus resulted in an allele determination. Based on the allelic profile for each sample, exact or approximate sequence types (STs) were assigned. Click here for file

Additional file 3: Figure S1 Dendrogram based on MLST from eight *S. epidermidis* samples. Samples identification numbers preceded by UFG are samples isolated in this study. Samples preceded by ST are from MLST database (http://sepidermidis.mlst.net/sql/download_st.asp). Click here for file
