# Supplementary material for: Molecular epidemiology of coagulase-negative Staphylococcus carriage in neonates admitted to an intensive care unit in Brazil
Source: BMC Infect Dis. 2013 Dec 5;13:572. doi: 10.1186/1471-2334-13-572 (PMC4028975; doi:10.1186/1471-2334-13-572)
Supplement: Additional file 1: Table S1 — SCCmec types and frequencies at admission and discharges among strains with more than one type isolated from neonates from July 2007 through May 2008. [file 1471-2334-13-572-S1.doc]

Table S1. SCC*mec* types and frequencies at admission and discharges among strains with more than one type isolated from neonates from July 2007 through May 2008.

|  | *S. epidermidis* | | *S. haemolyticus* | | *S. capitis* | | *S. warneri* | |
| --- | --- | --- | --- | --- | --- | --- | --- | --- |
| SCC*mec* type | Adm^*^ | Disch^**^ | Adm | Disch | Adm | Disch | Adm | Disch |
| I / II | 0 | 2 | 1 | 1 | 0 | 0 | 0 | 0 |
| I / III | 1 | 2 | 0 | 1 | 0 | 0 | 0 | 0 |
| I / IV | 0 | 4 | 0 | 0 | 0 | 0 | 0 | 0 |
| II / III | 2 | 6 | 1 | 1 | 0 | 0 | 0 | 0 |
| II / V | 0 | 12 | 0 | 0 | 0 | 0 | 0 | 0 |
| III / IV | 1 | 4 | 0 | 0 | 0 | 0 | 0 | 0 |
| III / V | 0 | 4 | 0 | 2 | 0 | 15 | 0 | 0 |
| IV / V | 0 | 1 | 0 | 2 | 0 | 0 | 0 | 0 |
| I / II / IV | 0 | 1 | 0 | 0 | 0 | 0 | 0 | 0 |
| I / III / IV | 0 | 2 | 0 | 1 | 0 | 0 | 0 | 0 |
| I / II / III / V | 0 | 1 | 0 | 0 | 0 | 0 | 0 | 0 |
| I / III / V | 0 | 0 | 1 | 1 | 0 | 0 | 0 | 0 |
| I / II / III | 1 | 0 | 2 | 2 | 0 | 0 | 0 | 0 |
| II / III / V | 0 | 8 | 0 | 0 | 0 | 0 | 0 | 0 |
| II / III / V | 0 | 0 | 0 | 0 | 0 | 0 | 0 | 0 |
| III / IV / V | 0 | 0 | 1 | 7 | 0 | 0 | 0 | 0 |

* Adm: Admission

** Disch: Discharge
