# Supplementary material for: Molecular epidemiology of coagulase-negative Staphylococcus carriage in neonates admitted to an intensive care unit in Brazil
Source: BMC Infect Dis. 2013 Dec 5;13:572. doi: 10.1186/1471-2334-13-572 (PMC4028975; doi:10.1186/1471-2334-13-572)
Supplement: Additional file 2: Table S2 — MLST analysis from eight S. epidermidis strains isolated from neonates in this study. Sequencing analysis of each locus resulted in an allele determination. Based on the allelic profile for each sample, exact or approximate sequence types (STs) were assigned. [file 1471-2334-13-572-S2.docx]

Table S2. MLST analysis from eight *Staphylococcus epidermidis* strains isolated from neonates in this study. Sequencing analysis of each locus resulted in an allele determination. Based on the allelic profile for each sample, exact or approximate sequence types (STs) were assigned.

| IDs^*^ | *arcC* | *aroE* | *gtr* | *mutS* | *pyr* | *tpi* | *yqIL* | Exact ST^**^ |
| --- | --- | --- | --- | --- | --- | --- | --- | --- |
| 119-D | 2 | 17 | 1 | 1 | 1 | 1 | 1 | 541^***^ |
| 420-A | 1 | 2 | 2 | 1 | 1 | 1 | 1 | 86 |
| 420-D | 57 | 1 | 1 | 2 | 1 | 1 | 1 | 542^***^ |
| 502-A | 1 | 2 | 6 | 2 | 22 | 1 | 10 | 543^***^ |
| 507-D | 2 | 17 | 1 | 1 | 2 | 1 | 1 | 81 |
| 608-A | 2 | 1 | 1 | 1 | 2 | 1 | 1 | 59 |
| 608-D | 2 | 1 | 1 | 1 | 2 | 1 | 1 | 59 |
| 665-A | 2 | 17 | 1 | 2 | 2 | 1 | 1 | 544^***^ |

* ID: identification number, A: admission, D: discharge

** Sequence type (ST) with exact match to all 7 loci

*** New ST assigned by the sepidermidis.mlst.net curator
