# Supplementary material for: Molecular epidemiology of coagulase-negative Staphylococcus carriage in neonates admitted to an intensive care unit in Brazil
Source: BMC Infect Dis. 2013 Dec 5;13:572. doi: 10.1186/1471-2334-13-572 (PMC4028975; doi:10.1186/1471-2334-13-572)

Additional file 3: Figure S1. Dendrogram based on MLST from eight *S. epidermidis* samples. Samples identification numbers preceded by UFG are samples isolated in this study (ST designations are in parenthesis). Samples preceded by ST are from MLST database (www.sepidermidis.mlst.net).


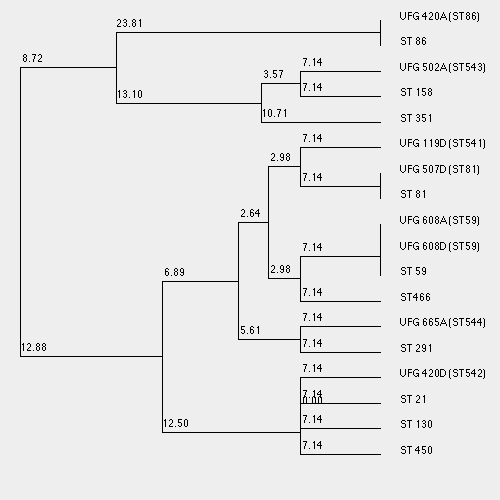

Supplement: Additional file 3: Figure S1 — Dendrogram based on MLST from eight S. epidermidis samples. Samples identification numbers preceded by UFG are samples isolated in this study. Samples preceded by ST are from MLST database (http://sepidermidis.mlst.net/sql/download_st.asp). [file 1471-2334-13-572-S3.doc]
